# *Chlamydia* beyond the genital tract: a major contributor to community-acquired pneumonia

**DOI:** 10.3389/fcimb.2026.1787885

**Published:** 2026-05-08

**Authors:** Siqi Tu, Yan Li, Yu Zhou, Zishan Zeng, Yanan Niu, Jin Lin, Jiaxin Chen, Jiaxuan Tang, Peng Liu, Yating Wen, Peng Ling, Jun Qu

**Affiliations:** 1 Institute of Pathogenic Biology, Basic Medical School, Hengyang Medical School, University of South China, Hengyang, China; 2 Department of Critical Care Medicine, The Central Hospital of Shaoyang City and Affiliated Shaoyang Hospital, Hengyang Medical College, University of South China, Shaoyang, China; 3 Radiology department, Affiliated Hengyang Hospital of Hunan Normal University &Hengyang Central Hospital, Hengyang, Hunan, China

**Keywords:** *Chlamydia pneumoniae*, *Chlamydia psittaci*, *Chlamydia trachomatis*, chlamydial pneumonia, community-acquired pneumonia

## Abstract

Chlamydial pneumonia is a respiratory infection caused by three distinct species of obligate intracellular pathogens that include *Chlamydia pneumoniae*, *C*. *psittaci*, and *C*. *trachomatis*. Although they differ morphologically and are transmitted by distinct routes, all three organisms can elicit a complex clinical spectrum that ranges from acute pneumonia to chronic inflammation and extrapulmonary disease. Globally, chlamydial pneumonia represents a significant proportion of community-acquired pneumonia (CAP) cases, with species-specific prevalence varying by population and diagnostic method. However, diagnosis can be challenging due to the non-specific nature of its clinical presentation. Although serological testing is available for laboratory diagnosis, empirical antibiotic therapy based on clinical assessment is often sufficient for cure. The clinical course is generally mild, and the disease responds well to appropriate antibiotic treatment. This review summarizes the etiology, epidemiology, pathogenesis, and clinical manifestations of chlamydial pneumonia. We critically evaluate current diagnostic and therapeutic approaches, and discuss the associated public health burden. By synthesizing the latest evidence across these domains, this analysis aims to advance scientific understanding and provide a robust foundation for optimizing prevention, diagnostic accuracy, and therapeutic strategies against these complex infections.

## 1 Introduction

Community-acquired pneumonia (CAP), a prevalent and severe respiratory infection, causes millions of cases annually worldwide with substantial hospitalization and mortality burdens. Particularly among the elderly, immunocompromised individuals, and patients with comorbidities, CAP escalates healthcare resource utilization and imposes major public health and socioeconomic pressures. Recent multinational studies based on large-scale epidemiological data indicate that precise pathogen identification and optimized therapeutic strategies are critical for reducing CAP-related mortality (Marchello et al., 2016; Martin-Loeches et al., 2023). Typical pathogens causing CAP include *Streptococcus pneumoniae*, *Hemophilus influenzae*, *Staphylococcus aureus*, and *Moraxella catarrhalis*; atypical pathogens include *Mycoplasma pneumoniae*, *Chlamydia pneumoniae*, and *Legionella pneumophila*.

The genus *Chlamydia* contains various species that can affect human animal health (Wang et al., 2019; Wan et al., 2025). They are Gram-negative, obligate intracellular prokaryotes that share a unique biphasic developmental cycle (Chen H. et al., 2022; Yang Y. et al., 2022). This cycle alternates between the infectious, environmentally resistant Elementary Body (EB) and the non-infectious, metabolically active Reticulate Body (RB) (Liu et al., 2022). EBs attach to host cells and enter via pinocytosis, then differentiate into RBs within a membrane-bound inclusion (Zhou Z. et al., 2024). RBs exploit host-derived energy and nutrients to multiply extensively by binary fission, ultimately re-differentiating into progeny EBs that are released to infect new cells, completing a 40-hour cycle. The EB’s outer membrane is reinforced by species-specific proteins, such as the Major Outer Membrane Protein (MOMP) (Wang B. et al., 2025), and cysteine-rich proteins, which enhance stability and infectivity (Banhart et al., 2019). These proteins are anchored in a double lipid bilayer (inner and outer membrane) that is characteristic of the genus. Division is atypical in that *Chlamydia*, which lacks the canonical bacterial cytokinesis organizer FtsZ, still divides by binary fission (Bayramova et al., 2018). Morphological details vary among species, as seen in the pear-shaped EBs of *C. pneumoniae* and the round EBs of *C. trachomatis*, differences that are thought to reflect distinct outer-membrane architectures (Kuo et al., 1995; Wen et al., 2020).

Among the atypical pathogens, *Chlamydia* species responsible for CAP mainly include *C. pneumoniae* (Zheng et al., 2015; Chen et al., 2017), *C. psittaci* (Wu et al., 2016; Sun et al., 2017), and *C. trachomati*s (Chen H. et al., 2020; Chen et al., 2021). Although emerging zoonotic species such as *C. pecorum* (Fukushi and Hirai, 1992) and avian *C. abortus* (Raven et al., 2025) have been occasionally implicated in human CAP cases, this synthesis primarily focuses on these three major pathogenic species (Szymańska-Czerwińska et al., 2023; Marti et al., 2024). Their non-specific clinical presentation frequently delays definitive diagnosis. Although serology remains the reference laboratory method, empirical antibiotic therapy guided by clinical assessment is usually curative and disease course is generally mild. Approximately 70% of *C. pneumoniae* infections are asymptomatic or lack typical symptoms, the remaining 30% progress to atypical CAP, bronchitis, or upper-respiratory-tract infection. Beyond the lungs, *C. pneumoniae* has been implicated in chronic inflammatory diseases including COPD, asthma, lung cancer, Alzheimer’s disease, multiple sclerosis, schizophrenia, atherosclerosis, and arthritis (Porritt and Crother, 2016), underscoring the need for early recognition and treatment to prevent systemic complications. Reports of *C. psittaci* pneumonia are rising; the infection can rapidly advance to severe pneumonia and respiratory failure, yet non-specific symptoms and limited sensitivity of conventional assays result in under-diagnosis and low clinical awareness (Deng et al., 2024). While *C. pneumoniae* and *C. psittaci* predominantly cause adult respiratory disease, *C. trachomatis* is a recognized pathogen of childhood pneumonia (Gautam and Krawiec, 2020).

Despite advances in understanding chlamydial biology and pathogenesis, translating this knowledge into improved clinical practice remains challenging. In this article, we systematically elaborate the definition, epidemiology, pathogenic mechanisms, and clinical significance of *C. pneumoniae*, *C. psittaci*, and *C. trachomatis*. It aims to provide a theoretical framework for developing broad-spectrum *Chlamydia* inhibitors, optimizing molecular diagnostic targets, and formulating stratified treatment strategies.

## 2 Epidemiological characteristics

### 2.1 Sources of infection and transmission routes

*C. pneumoniae* exclusively infects humans and transmits via respiratory routes through aerosols, droplets, and fomites (Theunissen et al., 1993; Gautam and Krawiec, 2020; Garin et al., 2022). Close contact with patients or self-inoculation of nasal/oral mucosa after touching fomites constitutes an additional route. Notably, *C. pneumoniae* can survive on inanimate surfaces for up to 30 h (Falsey and Walsh, 1993). While the population is universally susceptible, the probability of exposure and outbreak amplification rises sharply in crowded settings. Dormitories, correctional facilities, hospitals, long-term-care homes, military training centers and schools create networks of close proximity that facilitate propagation (Tagini et al., 2025).

*C. psittaci*, an obligate intracellular bacterium with broad host tropism, primarily originates from avian reservoirs (e.g., parrots, poultry). Human infection occurs through direct contact with fresh excreta (feces/urine) of infected birds, inhalation of respiratory/ocular secretions, or aerosolized dried excreta (Xie et al., 2023; Lu et al., 2024; Wu et al., 2024). Rare human-to-human transmission has been documented, involving not only close contacts of index cases but also secondary/tertiary cases and asymptomatic carriers within households (Zhang Z. et al., 2022; Cui and Meng, 2023). Importantly, although human-to-human transmission is rare, environmental exposure, such as inhalation of contaminated aerosols, can infect individuals without direct avian contact. A study from Lishui City, China, found that among four patients with *C. psittaci* pneumonia, two denied any history of avian contact. However, *C. psittaci* genotype E/B strains, identified by sequencing the outer-membrane-protein A (*ompA*) gene, were detected in their living environment, highlighting the importance of environmental transmission (Qin et al., 2022).

**Figure 1:**
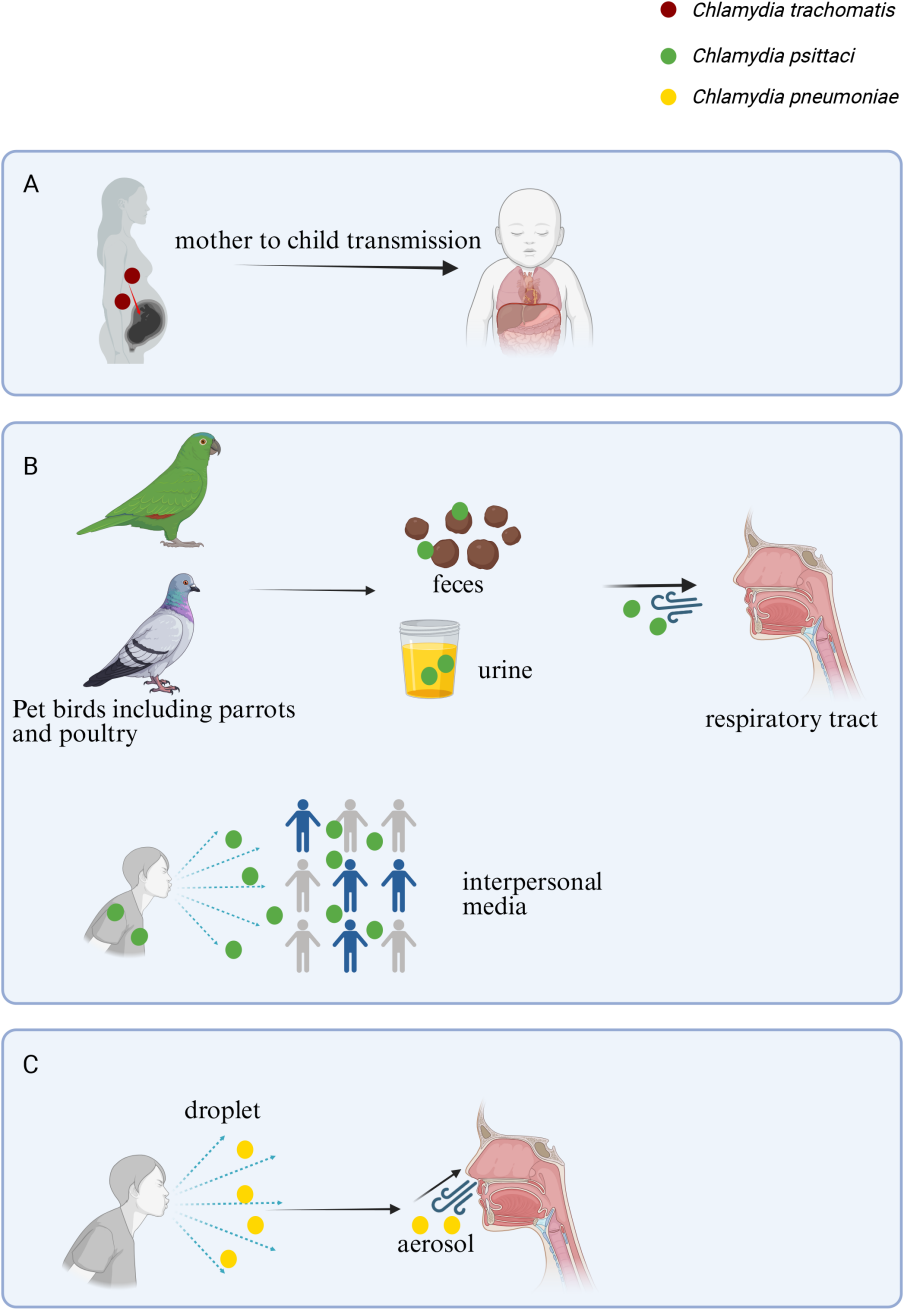
Infection sources and primary transmission routes of major pathogenic *Chlamydia* species. The three primary pathogenic *Chlamydia* species exhibit distinct host tropisms and transmission mechanisms. **(A)** *C. trachomatis* can cause neonatal pneumonia through vertical transmission. **(B)** *C. psittaci* originates from avian reservoirs, with human infection (psittacosis) occurring mainly through inhalation of aerosols from contaminated bird secretions or excreta; limited human-to-human transmission has been reported. **(C)** *C. pneumoniae* is a strictly human-adapted pathogen spread via respiratory droplets and aerosols. Created with BioRender.com.

*C. trachomatis* is primarily known for causing genitourinary infections (Zhang et al., 2025), which are most commonly spread through direct tissue contact via sexual routes (Yu et al., 2025), and a significant proportion of these infections remain asymptomatic (Grygiel-Górniak and Folga, 2023). Beyond the genitourinary tract, *C. trachomatis* can also lead to pneumonia, though this manifestation is relatively rare. Neonates are particularly vulnerable to *C. trachomatis* pneumonia, which typically occurs through vertical transmission from mothers who have chronic cervical infections caused by this pathogen (Mishra et al., 2011; Adachi et al., 2021; Mohseni et al., 2023). This mode of transmission highlights the potential for maternal infections to impact infant health, underscoring the importance of screening and treating genitourinary infections in pregnant individuals to prevent such outcomes (**Figure 1**).

### 2.2 Incidence rate

It is important to highlight that epidemiological data for chlamydial pneumonia are reported separately for each species due to their distinct transmission routes, affected populations, and diagnostic challenges. No consolidated prevalence estimate exists for all *Chlamydia* species combined in CAP. Globally, chlamydial pneumonia represents a significant proportion of community-acquired pneumonia (CAP) cases, with species-specific prevalence varying by population and diagnostic method (Camões et al., 2021; Garin et al., 2022; Murray et al., 2024).

*C. pneumoniae* is a significant pathogen of CAP, and recent epidemiological surveillance indicates that its incidence is demonstrating a marked resurgence. Large-scale PCR surveillance data from southern Germany revealed that the detection positivity rate surged from a baseline of 0.3% during 2015–2020 to 2.6% in 2024, remaining elevated at 2.4% through August 2025 (Wellinghausen et al., 2026); The nationwide surveillance network in China also reported a 4.3-fold increase in positivity rates in 2024 compared with 2022–2023 (from 0.21% to 0.90%) (Qin et al., 2025); The Marseille region in France even observed a 19-fold increase in diagnosed cases during 2024 (Edouard et al., 2025). In pediatric populations, the latest meta-analysis indicates that *C. pneumoniae* accounts for 1.1% (95% CI: 0.06%–3.0%) of severe pneumonia cases (Huang et al., 2025a). Collectively, these data suggest that *C. pneumoniae* is re-emerging as a significant clinical threat warranting heightened attention from the global public health community regarding its epidemiological characteristics.

Recently, pneumonia caused by *C. psittaci* has gained attention, with 1.03% of CAP cases annually attributed to it (Hogerwerf et al., 2017). Metagenomic next-generation sequencing (mNGS) analysis reveals it accounts for 8% of severe community-acquired pneumonia (SCAP) cases in immunocompetent ICU patients (Wu et al., 2020), compared to fewer than 50 annual US cases (Gautam and Krawiec, 2020). A Chinese study across 14 provinces found a 2.1% *C. psittaci* prevalence in complex/atypical pneumonia patients (2019–2021) (Huang W. et al., 2023). PCR detection in Dutch hospitals showed a 4.8% incidence, higher than previously reported (Spoorenberg et al., 2016). In Japan, *C. psittaci* IgM antibody testing in infants showed a 2.2% positive rate (Numazaki et al., 1992). These data suggest *C. psittaci* pneumonia incidence may be underestimated in epidemiological reports.

Sporadic *C. psittaci* pneumonia cases are rising globally, in both developed and developing countries, including Austria, Denmark, Germany (Olatunji et al., 2024), Sweden, the Netherlands (Saxena et al., 2025), Belgium (De Meyst et al., 2024), China, and Argentina (Favier et al., 2024; Hou et al., 2024; Qin et al., 2024). Since the first 1876 human outbreak report, Europe and the Americas experienced resurgences linked to exotic bird trade in the late 19th to early 20th centuries, detailed in Hegler’s 1930 research. A multicenter cohort in Argentina emphasized the occupational risk among animal workers, confirming its global zoonotic burden (Yang H. et al., 2024). Recently, with more household and farm birds, *C. psittaci* pneumonia outbreaks have become more frequent.

*C. trachomatis* pneumonia is a key cause of lower respiratory infections in neonates and infants, closely tied to mother-to-child vertical transmission. One study found 5%–30% of infants born to *C. trachomatis*-infected mothers develop pneumonia, typically detected between 4–12 weeks of age, though symptoms usually appear by 8 weeks (Mohseni et al., 2023). Another study shows that untreated maternal *C. trachomatis* infection can cause pneumonia in 10%–20% of infants, and *C. trachomatis*-positive infants have a 3.6-fold higher risk of perinatal pneumonia than negative ones (Arteaga-Troncoso et al., 2024). Although vaginal delivery is thought to be the main transmission route, 20% of infected cases are in cesarean-delivered infants, possibly due to intrauterine ascending infection or perinatal exposure to contaminated secretions. Notably, vaginally delivered infants have significantly higher *C. trachomatis* respiratory tract loads and peripheral blood leukocyte counts than cesarean-delivered infants, suggesting delivery mode may affect pathogen exposure and host immune response (Xu et al., 2018). A clinical cohort studies show *C. trachomatis* is detected in 30% of infants hospitalized for lower respiratory infections under six months old (Chen et al., 2007), with severe pneumonia accounting for 28.2% (Chen W. et al., 2024). In Kenya, *Chlamydia*-related pneumonia accounts for 51% of late-onset neonatal pneumonia, mainly presenting as interstitial pneumonia, with a prevalence eight times higher than in other regions, highlighting geographic epidemiological differences. Additionally, large-sample data indicates a 24.8% pneumonia incidence in *C. trachomatis*-infected newborns (Were, 2002), with a peak at six weeks postpartum but a wide time window of 1–19 weeks (Arteaga-Troncoso et al., 2024). These findings underscore the importance of prenatal screening and perinatal interventions in reducing disease burden.

### 2.3 High incidence population

#### 2.3.1 C. pneumoniae

The epidemiological characteristics of *C. pneumoniae* infection exhibit pronounced demographic distribution. Recent global surveillance and reviews confirm the continued impact of sex, age, and lifestyle on infection risk (Okami et al., 2024; Tagini et al., 2025). Regarding sex, multiple investigations report a higher prevalence among males. A Japanese cohort documented that males accounted for 58.8% of patients with mono-infection by *C. pneumoniae* and 63.2% of those co-infected with *Mycoplasma pneumoniae (*Oishi et al., 2020). A multivariable analysis conducted in the Netherlands identified male sex as an independent risk factor for *C. pneumoniae* infection, with men exhibiting a 1.7-fold higher risk than women (Raeven et al., 2016).

Age-stratified analyses reveal marked differences in the infection profiles of children and adults. A Japanese survey reported the highest seroprevalence (52.9%) among school-aged children 6–10 years (Oishi et al., 2020). Among adults and the elderly, divergent patterns emerge. A Jordanian study documented peak acute-infection rates of 25% in individuals aged 49–64 years (Al-Hajaya et al., 2020), whereas Dutch data demonstrated significantly higher prevalence in persons < 60 years compared with those ≥ 60 years (33.8% vs 12.4%) (Raeven et al., 2016). Of particular concern, European monitoring indicates a resurging trend in adult populations since 2024, emphasizing the need for continued age-specific surveillance (Tagini et al., 2025).

#### 2.3.2 C. psittaci

The epidemiological profile of *C. psittaci* pneumonia is intimately linked to host-exposure patterns and environmental determinants. *C. psittaci* infections exhibit pronounced age clustering, with adults aged 30–60 years representing the principal affected cohort; within this group, males markedly outnumber females (male-to-female ratio ≈ 1.6:1) (Shi et al., 2021). A study from central-south China that enrolled 116 laboratory-confirmed *C. psittaci* pneumonia patients documented a mean age of 59.7 years, with 79.3% of cases older than 50 years and 72.4% being male (Yang M. et al., 2022). This sex disparity is likely attributable to occupational exposure, as occupations such as poultry farming, slaughtering, and live-bird market work are disproportionately male-dominated. Of note, infections in children are infrequent. A Japanese serological investigation of 223 children hospitalized for pneumonia detected *C. psittaci*-specific IgM in only 2.2% of cases, indicating that infants and young children are not a high-risk population for *C. psittaci* infection (Numazaki et al., 1984). Moreover, data from European psittacosis outbreaks reveal a scarcity of pediatric cases (Olatunji et al., 2024).

*C. psittaci* infections display a bimodal age distribution: on the one hand, occupationally exposed individuals (e.g., poultry workers aged 30–60 years) constitute the primary reservoir because of sustained contact with infectious sources; on the other hand, immunocompromised or comorbid elderly patients (> 60 years) are more prone to develop severe pneumonia. In a 2019 cluster reported from Zhejiang Province, China, two affected women aged 76 and 64 years acquired infection following exposure to infected domestic fowl (Liu et al., 2023). Moreover, severe cases are concentrated in older adults. A study of 45 patients with SCAP reported a median age of 58 years, and 64.4% had documented poultry exposure (Tang et al., 2023).

Progression of *C. psittaci* pneumonia to critical illness is strongly associated with advanced age, underlying comorbidities, and delayed treatment. In severe cases the median PaO_2_/FiO_2_ ratio is only 119.8 mmHg, and the ICU mortality reaches 8.9% (Cai et al., 2016). Patients older than 60 years or those with hypertension or diabetes are more likely to develop multi-organ dysfunction, and the CD4^+^/CD8^+^ lymphocyte ratio correlates closely with disease severity. Although metagenomic next-generation sequencing (mNGS) has improved diagnostic yield, early recognition remains challenging because 30% of cases report no overt avian exposure (Tang et al., 2023).

#### 2.3.3 C. trachomatis

*C. trachomatis* pneumonia demonstrates marked age specificity and is primarily transmitted through mother-to-child vertical routes (Taylor-Robinson and Keat, 2015). Genital *C. trachomatis* prevalence among women of reproductive age is approximately 6–7%; without antenatal screening, roughly 50% of *C. trachomatis*-positive mothers transmit the organism during vaginal delivery, and 25–50% of infected neonates subsequently develop pneumonia or other clinical manifestations (Taylor-Robinson and Keat, 2015). A Mexican observation confirmed that the hazard rate of perinatal pneumonia was 3.6 times higher in *C. trachomatis*-positive babies than in *C. trachomatis*-negative babies (Arteaga-Troncoso et al., 2024). Post-acquisition, the pathogen ascends via the nasolacrimal duct to the respiratory tract, establishing persistent infection. Infants under two months, whose immunity is still immature, constitute the highest-risk group, and infection may persist for months or even years (Souza et al., 2012). Multicenter global data indicate that > 65% of *C. trachomatis* pneumonia cases occur in infants under six months, with peak onset at 1–4 months. For example, a Brazilian cohort of 151 infants detected *C. trachomatis* infection exclusively in those < 5 months, with the highest proportion (9.9%) within the first two months, and even caesarean-delivered infants were affected (20%) (Souza et al., 2012).

Age-specific incidence shows a steep, exponential decline. A retrospective study of 117 C*. trachomatis* pneumonia cases in Xiamen, China, found that 72.6% were infants < 3 months old, and 28.2% presented with severe pneumonia (Chen W. et al., 2024). Data from Ganzhou, China Maternal and Child Health Hospital demonstrated *C. trachomatis* infection rates of 7.19%, 4.95% and 1.19% among infants aged 1–3 months, 4 months–1 year and > 1 year, respectively (Numazaki et al., 1984). Japanese investigators using indirect immunofluorescence detected *C. trachomatis*-IgM antibodies in 29% of 109 infants with pneumonia, and viable organisms were isolated by McCoy cell culture from 66% of seropositive cases, confirming active early-life infection (Numazaki et al., 1984). Notably, *C. trachomatis* infection frequently co-occurs with other pathogens. 2.4% of Thai infants with acute bronchiolitis were co-infected, whereas an Indian study reported *C. trachomatis* in 22.8% of bronchiolitis cases < 6 months, most of whom had concurrent respiratory viral infections (Pientong et al., 2011). Recent literature has characterized *Chlamydia* as an “emerging old entity”, showing heterogeneous co-infection patterns globally, depending on regional pathogen circulation (Grygiel-Górniak and Folga, 2023). Additionally, Occupational exposure analyses further revealed that 81.25% of household members of *C. trachomatis*-infected infants worked in agriculture, forestry, animal husbandry, fisheries, commerce or catering, highlighting the potential for environmental exposure and intra-familial cross-transmission.

### 2.4 Seasonal distribution

#### 2.4.1 C. pneumoniae

Seasonal analyses across regions converge on a consistent pattern. *C. pneumoniae* infections peak during specific periods. A survey from Huzhou identified winter as having the highest detection rate (21.26%), followed by autumn (14.98%) (Gao et al., 2020). Conversely, Dutch data revealed significantly higher incidence during the non-respiratory season (May–October) than during the respiratory season (40.4% vs 14.2%) (Raeven et al., 2016). Notably, a Japanese study observed that *C. pneumoniae* prevalence was only 0.8% during the peak season of *Mycoplasma pneumoniae*, suggesting an inverse seasonal relationship between the two pathogens (Oishi et al., 2020). A global respiratory pathogen surveillance also confirmed this negative correlation pattern (Garin et al., 2022). Furthermore, underlying chronic diseases may influence infection risk. The same Dutch cohort reported a markedly increased risk among individuals without chronic obstructive pulmonary disease (COPD) (Raeven et al., 2016).

#### 2.4.2 C. psittaci

The seasonality of *C. psittaci* pneumonia exhibits marked geographic heterogeneity. In Europe, infections predominantly occur during the non-respiratory season (late spring to early autumn), coinciding with peak avian migration and breeding activities (Raeven et al., 2016). By contrast, Asian data reveal divergent patterns. Cases in Zhejiang Province cluster in autumn (September to November), whereas the Faroe Islands witness a September peak linked to traditional storm-petrel hunting, which is locally termed “September pneumonia” (Fossádal et al., 2018). A 2019–2021 multicenter Chinese study found the highest incidence in autumn (38.8%) and winter (33.6%), plausibly attributable to enhanced stability of aerosolized avian excreta under low-temperature conditions (Yang M. et al., 2022). Seasonal fluctuations in occupational exposure further modulate risk. For example, heightened bird trade in pet markets during winter months significantly elevates *C. psittaci* infection rates (Tolba et al., 2019). This seasonal risk is compounded by global climate variations affecting bird migration patterns and human-animal contact dynamics (Garin et al., 2022).

#### 2.4.3 C. trachomatis

The seasonal epidemiology of *C. trachomatis* infection exhibits marked geographic heterogeneity, with incidence peaks and underlying drivers varying substantially by region, climate, and sociocultural behavioral characteristics. Surveillance data from Shenzhen, China (2008–2019) showed monthly incidence rates ranging from 4.80 to 21.56 per 100,000 population, peaking in May and reaching a nadir in January–February (Weng et al., 2020). This fluctuation was not primarily driven by climatic factors, but was instead attributable to the “Spring Festival effect,” whereby reduced mobility among sexually active individuals and decreased healthcare-seeking behavior during the Chinese New Year holiday led to lower reported case numbers (Weng et al., 2020).

In contrast, studies from northern Vietnam (2016–2019) demonstrated a distinctly different pattern: co-infection rates of gonorrhea and *C. trachomatis* were significantly higher in summer (33.33%) than in winter (19.41%), suggesting a propensity for summer–autumn peaks in tropical monsoon climates (Tran et al., 2025). Laboratory surveillance data from England and Wales, United Kingdom (1991–2011), revealed fluctuations in weekly reported *C. trachomatis* cases without a consistent seasonal peak. These variations were closely linked to national screening strategies and sexual behavior patterns (Enki et al., 2013; Cherrie et al., 2018). In addition, studies of trachoma caused by *C. trachomatis* in Ethiopia associated seasonal fluctuations in ocular infection with deteriorating hygiene conditions due to water scarcity during the dry season, underscoring that climate-mediated environmental changes can strongly shape transmission efficiency in resource-limited settings (Greenland et al., 2022).

Collectively, available evidence indicates that seasonal fluctuations in *C. trachomatis* infection are driven primarily by the interplay of host behavioral factors, such as sexual activity patterns and holiday-related gatherings. And environmental exposures, including access to hygiene resources and climate-related changes. Regional differences in incidence peaks are thus fundamentally determined by the predominant local determinants (Enki et al., 2013; Cherrie et al., 2018; Weng et al., 2020; Tran et al., 2025).

Importantly, a nationwide registry project from Finland (2017) demonstrated that *C. trachomatis* infection in early life is strongly associated with mother-to-child vertical transmission, with risk closely correlated to maternal infection status. This finding suggests that the pathogenesis of severe *C. trachomatis*-related disease may operate independently of seasonal environmental influences, relying instead on host-specific factors such as perinatal vertical transmission (Honkila et al., 2017; Dzakah et al., 2021).

## 3 Clinical symptoms and diagnostic criteria

### 3.1 C. pneumoniae

Physical examination commonly reveals tachypnea, elevated temperature, and pharyngeal erythema with signs of chronic pharyngitis, otitis media, or sinusitis. Sputum is typically non-purulent. Auscultation detects only mild rhonchi, coarse crackles, or wheezing, which is discordant with radiographic infiltrates (Blasi et al., 2009; Gautam and Krawiec, 2020; Georgakopoulou et al., 2024). Primary infections often show unilateral alveolar infiltrates on imaging, whereas reinfections demonstrate bilateral pleural effusions and mixed infiltrates. Elderly patients may exhibit cardiomegaly or rare cavitation, with prolonged courses predisposing to secondary bacterial infections (Kauppinen and Saikku, 1995; Gaillat, 1996; Ma et al., 2022).

In laboratory tests, patients with pneumonia caused by *C. pneumoniae* have an average white blood cell count of 10.32 ×10^9^/L (compared to 9.87 ×10^9^/L in the control group), with 57.3% of patients having a normal white blood cell count. Their C-reactive protein (CRP) levels are 178.3 ± 129.81 mg/L, and aspartate aminotransferase (AST) levels are 33.93 ± 31.91 IU/L. Over one-third of patients show elevated AST and alanine aminotransferase (ALT) levels (Puljiz et al., 2006). And binary logistic regression analysis recently identified eosinophil count as a potential biomarker for severe *C. pneumoniae* pneumonia in children (p < 0.05) (Huang et al., 2025a).

### 3.2 C. psittaci

Clinical manifestations of *C. psittaci* pneumonia are highly non-specific and frequently overlap with other acute respiratory infections. Typical presentation includes abrupt high fever (median temperature 39.7 °C) (Knittler and Sachse, 2015), chills, headache and generalized myalgia (64.5% in the severe group) (He et al., 2023), with approximately 71% of patients reporting a dry cough (Knittler and Sachse, 2015). Chest pain and hemoptysis are uncommon (Deng et al., 2024). Notably, dyspnea is a critical early warning sign of severe pneumonia, occurring in 100% of the severe cohort and significantly more often than in the non-severe group (Knittler and Sachse, 2015). In rapidly progressive cases, multilobar involvement can develop within days, with 86.9% of severe cases affecting ≥ 2 lobes (Deng et al., 2024). Imaging typically reveals subpleural consolidation (61.3%) (He et al., 2023) accompanied by air bronchograms (5/5 cases) (Kong et al., 2021), often co-existing with ground-glass opacities (GGO) and pleural effusion (35.5%) (Deng et al., 2024). Necrotic cavitation and tree-in-bud patterns are rare (Wu et al., 2023) features that help distinguish psittacosis from bacterial pneumonia.

Laboratory findings reveal a “dissociation of inflammatory indices” in which leukocyte counts are usually normal or only mildly elevated (only 2/5 in the severe group showed elevation), whereas C-reactive protein (CRP) and procalcitonin (PCT) are markedly deranged (Kong et al., 2021). In severe cases CRP frequently exceeds 100 mg/L (abnormal in 85.2%, and 100% in non-survivors) and correlates significantly with mortality (Deng et al., 2024). Coagulation parameters show prolonged APTT and elevated D-dimer levels, indicating coagulation-fibrinolysis imbalance. Thrombocytopenic trends may further exacerbate organ injury. Cardiac biomarkers, which include Hypersensitive troponin I (HS-TNTI ≥ 50 ng/L) and myoglobin (MYO ≥ 300 ng/mL), are significantly elevated in fatal cases (Yang F. et al., 2022), underscoring myocardial involvement and its association with poor prognosis. Reduced arterial partial pressure of oxygen (PaO_2_) and multilobar infiltrates are key indicators for disease severity (Deng et al., 2024). Although lymphopenia (93% in severe cases) and hyponatremia (100%) are common (Kong et al., 2021; Yang F. et al., 2022), demographic variables such as age, sex or smoking history do not differ significantly (Yang F. et al., 2022). Notably, 35.5% of severe cases involved only a single lobe (11/31), indicating that radiographic extent alone is not an absolute determinant of severity (He et al., 2023). Early recognition of rapidly rising CRP/PCT values (ahead of leukocyte changes) and declining oxygenation indices, together with positive metagenomic next-generation sequencing (mNGS) results (75% in non-survivors), is critical for timely intervention (Knittler and Sachse, 2015; Yang F. et al., 2022).

### 3.3 C. trachomatis

Clinical presentation of *C. trachomatis* pneumonia is dominated by infants, the majority being < 6 months old (median age 2.5 months). No significant sex difference is observed, although males are slightly more frequently affected. Approximately 30% of infants hospitalized for pneumonia test positive for *C. trachomatis (*Chen et al., 2007). The classic picture is sub-acute afebrile pneumonia. 72–90% of cases are apyrexial, with hallmark paroxysmal cough (only 22% exhibit “staccato” cough) and tachypnoea (83%). A prodromal phase with coryzal symptoms is common (67%, lasting a mean of 7 days), and one-third present with concomitant conjunctivitis (33%) (Chiang et al., 2005; Chen et al., 2007). Pre-term infants may experience apneic episodes. Auscultation reveals crackles, whereas wheeze is unusual (Mohseni et al., 2023).

Laboratory findings include peripheral blood eosinophilia (≥ 400 cells/mm³) in 47% of cases, which correlates significantly with elevated specific IgM antibodies in term infants and serves as an ancillary diagnostic marker (Chen et al., 2007; Arteaga-Troncoso et al., 2024). Chest radiographs typically show bilateral interstitial infiltrates, pulmonary hyperinflation and, rarely, a miliary reticulonodular pattern that must be distinguished from tuberculosis (Camlar et al., 2009).

Among predictors of severe disease, a white blood cell count > 15,000 cells/μL and elevated PaCO_2_ independently increase risk, whereas congenital heart disease and mixed infection are associated with lower risk (Chen W. et al., 2024) (**Table 1**).

**Table 1:** Comparative clinical, radiological and laboratory features of *C. pneumoniae*, *C. psittaci* and *C. trachomatis* respiratory infections.

Symptom/diagnostic criteria	*C. pneumoniae*	*C. psittaci*	*C. trachomatis*
Fever	●Low-grade fever	● Sudden high fever (median 39.7 °C)	△Afebrile or low-grade (72%–90% no fever)
Cough	●Persistent dry cough (progresses over 1–4 weeks)	●Severe dry cough (71% cases)	●Paroxysmal cough (22% characteristic “staccato cough”)
Respiratory Symptoms	●Pharyngitis/laryngitis/sinusitis (initial phase) ●Tachypnea	● Dyspnea (100% in severe group)	●Tachypnea (83%) ●Prodromal rhinorrhea (67%, lasts 7d)
Imaging Features	●Unilateral alveolar infiltration (initial) ●Bilateral pleural effusion (reinfection) △Cardiomegaly/cavitation (elderly)	●Multilobar infiltration (86.9% severe) ●Subpleural consolidation and air bronchogram ●GGO/pleural effusion	●Bilateral interstitial infiltration ●Pulmonary hyperinflation △Miliary reticulonodular opacities (rare)
Risk Factors/Prognosis	△ Prolonged course (prone to bacterial superinfection)	●↓Oxygenation index ●↑Myocardial injury markers (high mortality risk) ● mNGS positivity (75% non-survivors)	●PaCO_2_↑ △↓Congenital heart disease
Laboratory Findings	△Normal WBC △Mildly↑CRP	●CRP>100mg/L (85.2%severe) ●PCT↑ ●Lymphopenia (93% severe)	●↑Eosinophils (47%) ●↑IgM antibodies (term infants)
Special Populations	●Elderly: mixed infiltrates	●Avian exposure (key diagnostic clue)	●Predominantly infants <6mo (median 2.5mo) ●Apnea risk in preterm neonates

●: Core/typical manifestation; △: Occasional/non-specific.

### 3.4 Pathogenesis

As an obligate intracellular pathogen, the pathogenicity of *Chlamydia* relies on a series of sophisticated molecular mechanisms to successfully achieve cellular invasion, intracellular survival, replication, and immune evasion (Chen et al., 2019; McCullough et al., 2025). Its pathogenic cycle begins with the injection of effector proteins via the Type III Secretion System (T3SS), which manipulate host actin cytoskeleton rearrangement for internalization and hijack the host’s lipid trafficking systems to meet its biosynthetic demands. Inside the cell, *Chlamydia* ensures a stable replicative niche by persistently activating pro-survival signaling pathways and inhibiting apoptosis. Concurrently, the pathogen employs multi-layered immune evasion strategies, including downregulation of antigen presentation, interference with immune effector molecules, and induction of an immunosuppressive state. These highly coordinated mechanisms collectively form the foundation for *Chlamydia*’s successful establishment of intracellular infection. While *C. pneumoniae* is a major cause of CAP; *C. psittaci* causes psittacosis (avian chlamydiosis) through inhalation of contaminated aerosols (Chen et al., 2023); and *C. trachomatis* primarily causes ocular and genital infections (Zhou et al., 2019), it can also cause pneumonia in infants through perinatal transmission. Each species employs species-specific strategies adapted to pulmonary infection, collectively forming the foundation for successful establishment of respiratory tract infection.

## 4 Pathogenic mechanism

### 4.1 Chlamydial invasion

**Figure 2:**
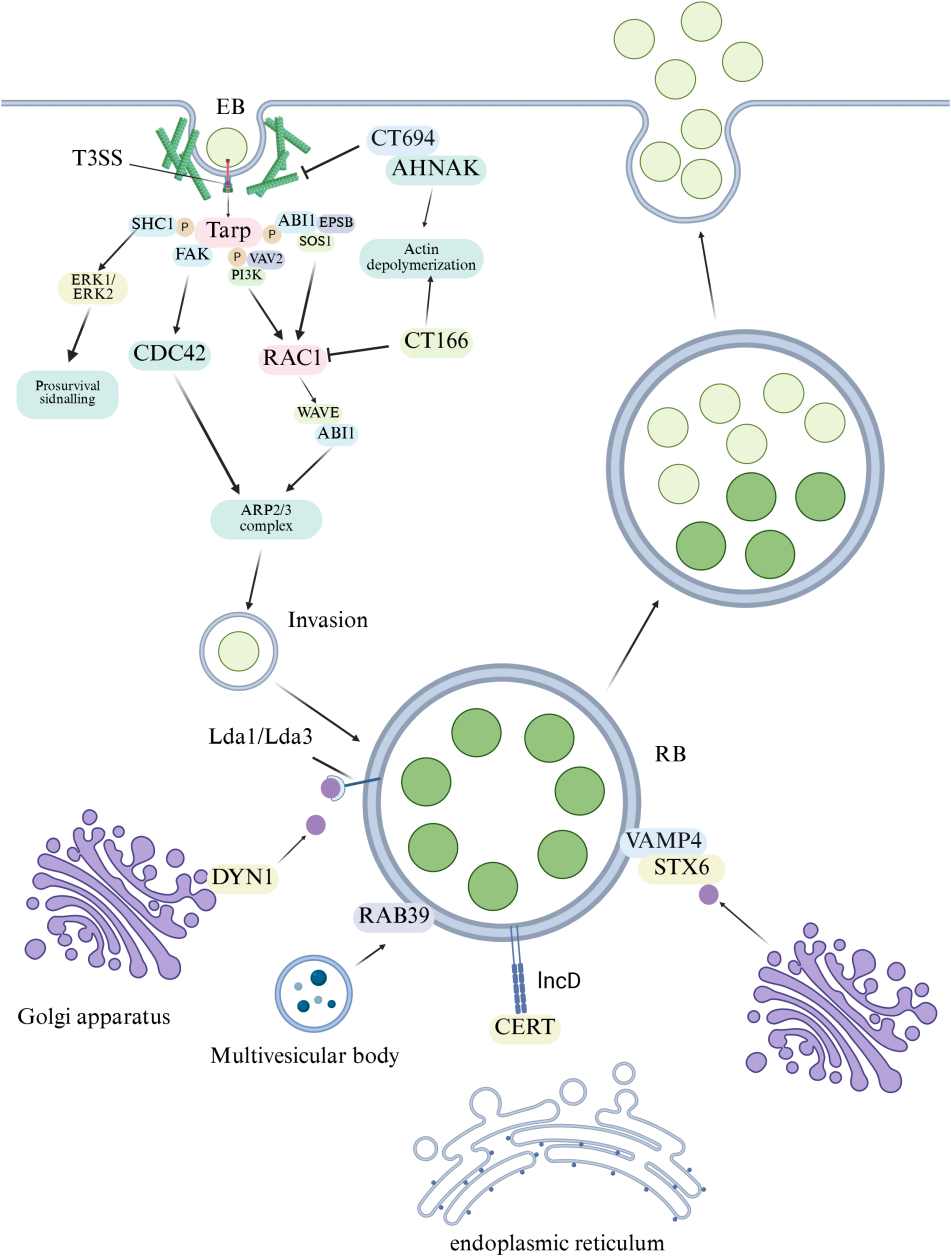
Molecular mechanisms of chlamydial host cell invasion and lipid acquisition. During invasion, the pathogen utilizes a Type III Secretion System (T3SS) to inject effector proteins (e.g., TarP, CT694). Phosphorylated TarP acts as a central hub, recruiting host adaptor proteins (SHC1, SOS1-ABI1-EPS8) and activating signaling pathways (PI3K, VAV2). CT694 coordinates with these activated pathways to regulate the RAC1/CDC42 pathway, leading to activation of the WAVE-ABI1 and ARP2/3 complexes, and subsequent actin cytoskeleton remodeling for bacterial internalization. CT166 promotes actin depolymerization by inactivating RAC1. For lipid acquisition, *Chlamydia* employs vesicular (hijacking RAB39/VAMP4-positive multivesicular bodies) and non-vesicular (via IncD binding the ceramide transfer protein CERT) pathways to secure essential lipids from the host, supporting intracellular replication and inclusion biogenesis. Created with BioRender.com.

During the invasion stage, the pathogen injects pre-synthesized effector proteins (such as TarP, CT694, TepP) via the Type III Secretion System (T3SS). Phosphorylated TarP plays a central role (Dai and Li, 2014). It recruits and activates various host factors including SHC1, VAV2, the SOS1-ABI1-EPS8 complex, and PI3K (Phosphoinositide 3-Kinase). This action, coordinated with the interaction between CT694 and AHNAK, precisely regulates the RAC1/CDC42 signaling pathway (Pais et al., 2013; Elwell et al., 2016). This subsequently activates the WAVE-ABI1 and ARP2/3 (Actin-Related Protein 2/3) complexes, driving the rearrangement of the actin cytoskeleton to complete internalization (Jiwani et al., 2013). Simultaneously, CT166 balances actin depolymerization by inactivating RAC1 (Bastidas et al., 2013) (**Figure 2**).

*C. pneumoniae* invasion of respiratory epithelium involves T3SS effector SemD, which recruits phosphatidylinositol 4-kinase IIIβ to produce phosphatidylinositol 4-phosphate, creating a favorable replicative niche (Kocher et al., 2024). CopB forms pores in the host cell membrane for effector translocation (Bulir et al., 2015), while the Pmp protein family (21 members) mediates adhesion to respiratory epithelial cells through repetitive FxxN and GGA (I,V,L) motifs, triggering inflammatory responses (Mölleken et al., 2010; Debrine et al., 2023) (**Figure 2**).

*C. psittaci* employs distinct adhesins for respiratory infection, including outer membrane protein OmcB, which mediates initial binding to host cell glycosaminoglycans, and Polymorphic Membrane Proteins Pmp22D, Pmp8G, and Pmp17G, which recognize epidermal growth factor receptor (EGFR) to activate intracellular internalization (Favaroni et al., 2021; Li et al., 2022). Pmp17G exhibits time-dependent adhesion kinetics, promoting bacterial binding in an EGFR-dependent manner at 120 minutes post-treatment (Li et al., 2022). Protein disulfide isomerase (PDI) also participates in *C. psittaci* entry into respiratory cells (Abromaitis and Stephens, 2009). The T3SS translocon proteins sctW and IncA associate with the inclusion membrane, controlling vesicle fusion and trafficking essential for pulmonary infection (Beeckman et al., 2008) (**Figure 2**).

Nascent inclusions of *C. trachomatis* and *C. pneumoniae* migrate along microtubules to the microtubule-organizing center (MTOC), a process requiring microtubule polymerization, dynein motor proteins, and Src family kinases. This transport facilitates nutrient acquisition and homotypic fusion of inclusions (Kokes and Valdivia, 2012; Tan and Bavoil, 2012). This process is independent of p50 dynamitin, suggesting that bacterial effectors may directly anchor inclusions to dynein or centrosomes. Four inclusion membrane proteins (IncB, CT101, CT222, and CT850) localize to centrosome-inclusion contact sites and co-localize with activated SFKs, implicating these proteins in the transport process (Kokes and Valdivia, 2012; Tan and Bavoil, 2012). CT850 of *C. trachomatis* directly interacts with the dynein light chain DYNLT1 (Tctex1), promoting inclusion positioning at the MTOC (Mital et al., 2015). IncB of *C. psittaci* associates with the host protein Snapin, which interacts with both SNARE proteins and dynein, suggesting that the IncB-Snapin complex may link inclusions to the dynein complex to drive transport (Böcker et al., 2014; Elwell et al., 2016) (**Figure 2**).

As an obligate intracellular pathogen, *Chlamydia* lacks biosynthetic enzymes for key membrane lipids (such as cholesterol, sphingomyelin, and specific phospholipids) and must therefore rely on host cell sources to meet its membrane biogenesis and proliferation needs. Its mechanisms for lipid acquisition are primarily divided into vesicle-mediated and non-vesicle-mediated pathways (Elwell and Engel, 2012). In the vesicle pathway, *Chlamydia* hijacks host cell vesicular trafficking by modulating factors including ARF (ADP-ribosylation factor) GTPases (Böcker et al., 2014), its guanine nucleotide exchange factor GBF1 (Golgi-specific BFA resistance factor 1), RAB family GTPases (such as RAB39) (Damiani et al., 2014), and VAMP4 (Vesicle-Associated Membrane Protein 4) (Kabeiseman et al., 2013). This redirects lipid-rich intracellular carriers, such as multivesicular bodies, to the inclusion membrane for fusion, enabling direct lipid acquisition. In the non-vesicle pathway, *Chlamydia* utilizes its inclusion membrane protein IncD to directly bind the host ceramide transfer protein (CERT), facilitating the direct transport of endoplasmic reticulum-derived ceramide to the inclusion (Derré et al., 2011; Wen et al., 2025). Subsequently, recruited host sphingomyelin synthase 2 (SMS2) converts this ceramide into sphingomyelin (Elwell et al., 2011). These mechanisms collectively ensure a continuous supply of lipids during intracellular parasitism, supporting bacterial replication and inclusion stability (**Figure 2**).

### 4.2 Chlamydial immune evasion

**Figure 3:**
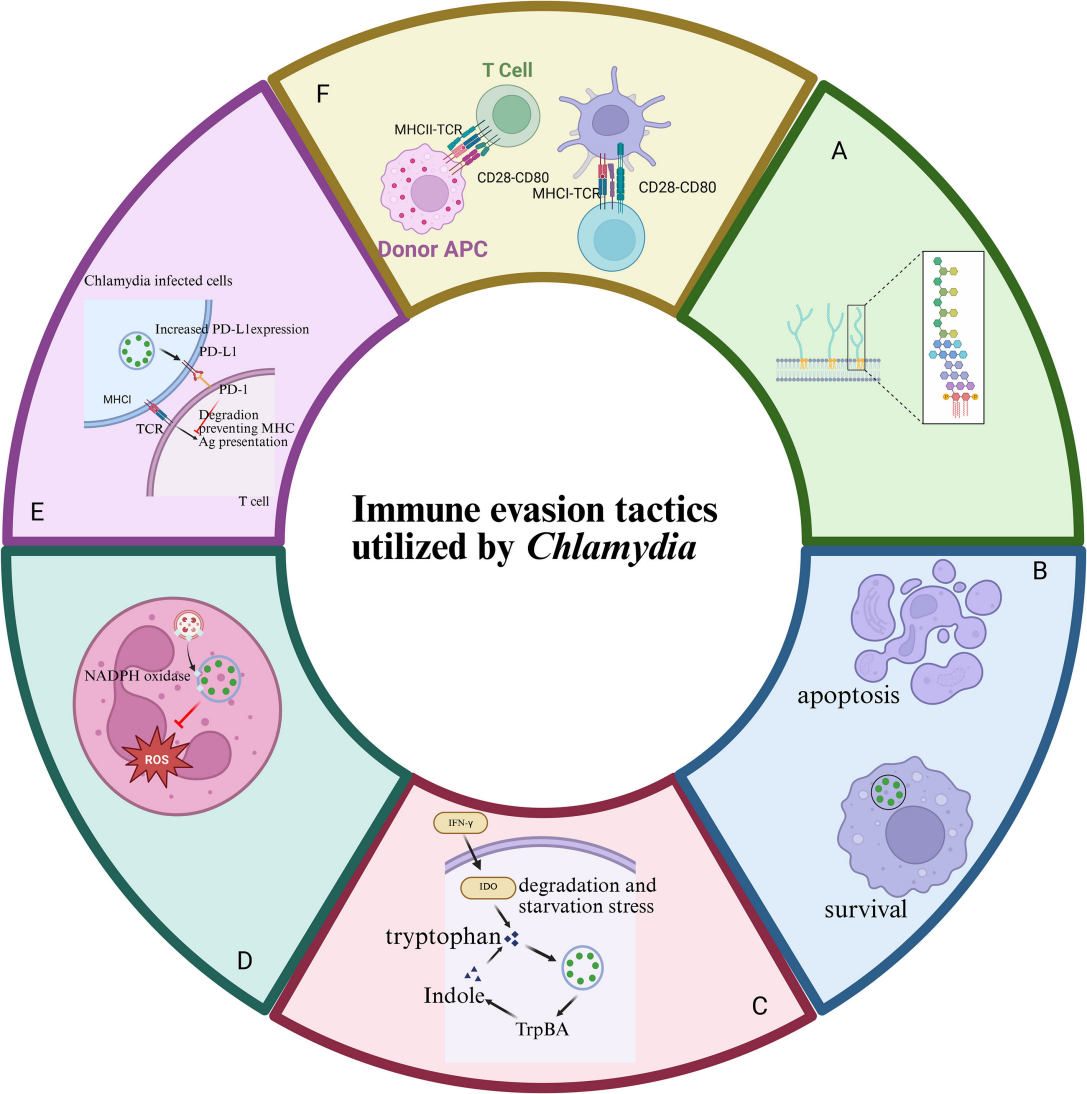
Multifaceted immune evasion strategies of *Chlamydia*. **(A)** Chlamydial LPS and LOS evade host complement system recognition, reducing inflammatory responses. The unique structure of chlamydial LPS, with limited glycosylation and long acyl chains, avoids PRR recognition. **(B)** *Chlamydia* regulates host cell apoptosis to support intracellular survival. It inhibits apoptosis early by blocking pro-apoptotic pathways and activating pro-survival signals, but can promote apoptosis later for dissemination. **(C)** *C. trachomatis* counters IFN-γ-induced tryptophan depletion by entering a tryptophan-independent state and secreting tryptophan synthase TrpBA. This allows the bacteria to resume replication when IFN-γ levels decline. **(D)** *Chlamydia* inhibits the production of key bactericidal molecules like ROS and NO by relocating NADPH oxidase subunits, reducing phagocyte killing efficiency. **(E)** *Chlamydia* degrades MHC molecules via CPAF protease and upregulates PD-L1 on host cells. This suppresses T cell activation and enables multi-pathway immune evasion. **(F)** *C. trachomatis* evades the host immune system by downregulating MHC-I and MHC-II expression, hindering CD8^+^ and CD4^+^ T cell activation. Created with BioRender.com.

*Chlamydia*’s structurally unique LPS (Lipopolysaccharide) and LOS (Lipooligosaccharide) evade recognition and activation of the host complement system, reducing inflammatory responses (Yang C. et al., 2019). In the chlamydial genome, the 2-8, 2–4 linked tri-Kdo (3-deoxy-D-manno-oct-2-ulopyranosonic acid) and the absence of LPS heptosyltransferase I (encoded by waaC) prevent further glycosylation (Yang C. et al., 2019). Consequently, chlamydial LPS has a deeply rough Re phenotype, avoiding Pattern Recognition Receptor (PRR) recognition. The substitution at the R3′ position of Kdo3-lipid IVA with an (R)-3-hydroxymyristoyl chain prevents further acylation (Yang C. et al., 2019). The amide-linked acyl and 3′ O-linked acyl chains (R2, R2′, R2″) on chlamydial lipopolysaccharide are unusually long (C18–20 compared to C12-14) (Yang C. et al., 2019). *Chlamydia* regulates host cell apoptosis to support its intracellular survival. Initially, it inhibits apoptosis by blocking pro-apoptotic pathways and activating pro-survival signals (Wen et al., 2021; Liao et al., 2025). For example, *C. psittaci* Inc proteins CPSIT_0556 and CPSIT_0846 suppress apoptosis in human neutrophils and HeLa cells (He et al., 2022), respectively, through pathways like PI3K/Akt and NF-κB (Chen Q. et al., 2020; He et al., 2021). Pgp3 protein of *C. trachomatis* inhibits apoptosis via HO-1 upregulation mediated by PI3K/Akt activation (Shu et al., 2023). *C. pneumoniae* not only subtly harnesses host inflammatory pathways, such as NLRP3 inflammasome activation, to achieve optimal intracellular growth, but can even hijack the autoregulatory loop of IL-1β to promote its own survival (Itoh et al., 2014; Koronyo-Hamaoui et al., 2025). However, in later stages, *Chlamydia* can also promote apoptosis to facilitate dissemination (Wang X. et al., 2025). The *C. psittaci* inclusion protein CPSIT_0842, for instance, induces macrophage apoptosis via the MAPK/ERK/mTOR pathway by triggering incomplete autophagy (Huang Y. et al., 2023), and the unfolded protein response via the PERK and IRE1α to regulate human bronchial epithelial cell autophagy after *C. psittaci* infection (Chen L. et al., 2022; Luo et al., 2023). CYTOR/miR-206/MAPK1 axis was involved in the regulation of autophagy in *C. trachomatis* infection (Cheng et al., 2024). When immune cells secrete IFN-γ (Interferon-gamma), inducing IDO (Indoleamine 2,3-dioxygenase) expression and tryptophan degradation in an attempt to starve the pathogen (Taylor and Feng, 1991; Thomas et al., 1993), *C. trachomatis* and *C. pneumoniae* not only can enter a tryptophan-independent “persistent” state to avoid immune surveillance (Beatty et al., 1994), but also actively compensates for tryptophan depletion by secreting tryptophan synthase TrpBA (whose α subunit converts IGP [Indole-3-glycerol phosphate] to indole, and β subunit synthesizes tryptophan from indole) (Mpiga and Ravaoarinoro, 2006; McClarty et al., 2007). The bacteria resume active replication when IFN-γ levels decline, leading to recurrent infection (Fan et al., 2002; Chen et al., 2025). In countering innate immunity, *Chlamydia* inhibits the production of key bactericidal molecules like ROS (Reactive Oxygen Species), NO (Nitric Oxide), and downstream MPO (Myeloperoxidase) and HOCl^-^ (hypochlorite) by relocating NADPH oxidase subunits to the inclusion membrane instead of the phagolysosome membrane (Boncompain et al., 2010; Marangoni et al., 2014; Chen et al., 2019), significantly reducing the killing efficiency of phagocytes. Furthermore, *Chlamydia* degrades MHC molecules via the CPAF (Chlamydial Protease/Proteasome-like Activity Factor) protease to block antigen presentation and simultaneously upregulates the expression of PD-L1 (Programmed Death-Ligand 1) on host cells (Wong et al., 2019). The binding of PD-L1 to PD-1 (Programmed Cell Death Protein 1) on T cells delivers an inhibitory signal, collectively suppressing T cell receptor (TCR)-mediated activation. These mechanisms ultimately enable multi-pathway, multi-layered immune evasion (Wong et al., 2019). *C. trachomatis* employs a highly coordinated set of mechanisms to evade the host immune system. It directly impairs the core adaptive immune response by downregulating the expression of MHC-I (Major Histocompatibility Complex Class I) (Zhong et al., 1999) and MHC-II (Major Histocompatibility Complex Class II) (Wolf and Fields, 2013) molecules on host cells, thereby hindering the activation of CD8^+^ cytotoxic T lymphocytes (CTLs) and CD4^+^ T helper (Th) cells (**Figure 3**).

## 5 Diagnostic methods

### 5.1 C. pneumoniae

#### 5.1.1 Microbiological culture

Microbiological diagnosis relies on isolating the pathogen from nasopharyngeal, pharyngeal swab, sputum, or pleural fluid samples. For diagnostic purposes, nasopharyngeal swabs and sputum samples represent the most frequently utilized respiratory specimens. Nasopharyngeal swabs are considered valid specimen types, often demonstrating diagnostic yields similar to those of sputum (Tagini et al., 2024). Nevertheless, the pathogen load (copy numbers per mL) can vary substantially depending on the stage of infection. Consequently, lower respiratory tract specimens are recommended when sampling occurs during the later stages of the illness. In specific scenarios, particularly involving severe illness or immunocompromised status, more invasive procedures yielding samples like bronchoalveolar lavage fluid or bronchial aspirates may be employed for analysis (Tagini et al., 2025). As it cannot grow in cell-free media, it is typically cultured in HEp-2 or HL cell lines for 4–7 days. When sampling, it is recommended to use polyethylene swabs and store specimens in sucrose phosphate buffer containing antibiotics, and after 72 hours of culture, confirm using species or genus-specific fluorescent antibodies, combined with *C. trachomatis* staining to rule out psittacosis (Hammerschlag, 2003).

#### 5.1.2 Serological diagnosis

Serological diagnosis of *C. pneumoniae* primarily employs microimmunofluorescence (MIF) and enzyme immunoassay (EIA) (Dowell et al., 2001). MIF, the consensus reference standard, uses formalinized elementary bodies to detect species-specific IgM, IgA, or IgG (Vainas et al., 2003), with diagnostic criteria requiring either a ≥ 4-fold rise in IgG/IgA titers between paired sera or single-sample IgM ≥ 1:16 and/or IgG ≥ 1:512 (Tagini et al., 2025). However, IgM emerges 2–3 weeks post-symptom onset and remains undetectable in early acute infection or reinfection (Hvidsten et al., 2009), while convalescent sampling necessitates 4–6 week follow-up, constraining therapeutic utility (She et al., 2010; Puolakkainen, 2013). The retrospective nature of diagnosis carries substantial risk of missed infections, and cross-reactivity with other *Chlamydia* species compromises specificity (Verkooyen et al., 1998).

EIA offers a more practical, objectively interpretable alternative with excellent intra-test consistency and high concordance with MIF at low titers. Automated ELISA platforms (e.g., SeroCP, SeroCP Quant) demonstrate specificities ≥ 99% and correlate well with MIF titers (Hermann et al., 2004), though sensitivity diminishes for low-titer samples, which is a limitation shared by both methodologies (Hermann et al., 2004). Neither MIF nor EIA effectively discriminates acute from persistent infection, as IgG persistence merely reflects prior exposure (Puolakkainen, 2013), while sustained IgA elevation has been proposed but not validated as a marker of chronicity (Verkooyen et al., 1998; Burillo and Bouza, 2010).

Complement fixation is discouraged due to poor sensitivity and genus-wide reactivity (Verkooyen et al., 1998; Burillo and Bouza, 2010). Consequently, serology remains valuable for epidemiological studies but limited for acute clinical decision-making, prompting the predominance of PCR-based diagnostics for initial patient management (Thurman et al., 2011).

#### 5.1.3 Molecular biological detection

DNA amplification techniques, such as PCR, are considered the most promising methods for developing rapid, non-culture-based detection of *C. pneumoniae*. However, current test results show significant variability. This is due to differences in specimen collection, processing, primer design, nucleic acid extraction, and product detection. PCR positivity varies from 0%–60% across labs, with some reporting false positives in negative controls, especially with nested PCR (Hammerschlag, 2003). Despite limitations of serology and culture, PCR analysis of respiratory samples has become the preferred method. Multiplex PCR can assess multiple pathogens without significantly reducing sensitivity. In 2012, the U.S. Food and Drug Administration (FDA) approved the FilmArray Respiratory Panel for detecting *C. pneumoniae* and other microbes in nasal swabs (Hammerschlag et al., 1992). However, PCR still faces specificity challenges due to asymptomatic carriage and persistence post-antibiotic treatment. Positive results could indicate persistent infection, reinfection, or asymptomatic carriage, and detecting *C. pneumoniae* in respiratory samples doesn’t rule out co-infection (Verkooyen et al., 1998; Jain et al., 2015; Loens and Ieven, 2016). Some techniques, like detecting circulating Chlamydial lipopolysaccharide, remain confined to research due to complexity (Gasparrini et al., 2020). In pediatric community-acquired lower respiratory tract infections, serology and nested PCR each have diagnostic utility, but nested PCR’s sensitivity is lower with a limited positive predictive value (Kumar et al., 2020).

The diagnostic landscape for *C. pneumoniae* has undergone revolutionary changes over the past decade, with qualitative leaps in speed, sensitivity, and convenience. Multiplex PCR and next-generation sequencing have become integral components of routine diagnostics for CAP. The U.S. FDA has approved multiple commercial multiplex PCR panels, such as the QIAstat-Dx RP2.0 and Luminex NxTAG RPP, both of which include detection of *C. pneumoniae* and *Mycoplasma pneumoniae*, enabling rapid parallel screening for multiple respiratory pathogens (Laves, 1975; Popowitch et al., 2022). These platforms significantly reduce diagnostic turnaround time, providing a prerequisite for targeted therapy. Furthermore, CRISPR/Cas12a-based detection technologies demonstrate substantial potential (Sinan et al., 2025). These approaches combine the high sensitivity of recombinase polymerase amplification (RPA) or isothermal linear amplification (ERA) with the high specificity of CRISPR systems, enabling rapid, visual detection of target nucleic acids without the need for expensive instrumentation (Zhou Y. et al., 2024). For *C. pneumoniae*, researchers have successfully developed RPA-CRISPR/Cas12a and ERA-CRISPR/Cas12a assays, with sensitivities as low as 2 copies per reaction and no cross-reactivity (Chen J. et al., 2022; Ling et al., 2023; Zhu et al., 2026).

#### 5.1.4 Imaging examinations

The imaging manifestations of *C. pneumoniae* pneumonia are non-specific, and chest X-ray and CT scans provide important references for diagnosis but lack etiological certainty. Chest X-rays often show unilateral alveolar infiltration or consolidation, predominantly in the lower lobes (Sharma et al., 2016), with about 88.76% of cases presenting interstitial infiltration and only 5.62% showing segmental consolidation or bronchopneumonia (Puljiz et al., 2006). In the early stages of the disease, chest X-rays typically display unilateral lesions, but follow-up examinations after 3.8 days may reveal progression to bilateral involvement (McConnell et al., 1994). The incidence of pleural effusion is relatively low (3.36%–25%), and hilar or mediastinal lymphadenopathy is rare. Imaging differences may be related to the timing of the examination, diagnostic methods, and co-infections (Nambu et al., 2006; Sharma et al., 2016). On CT scans, ground-glass attenuation (n = 38) and acinar pattern changes (n = 28) are the main features, with the latter, though non-specific, being of suggestive value (Ogawa et al., 2005). Due to the high variability in imaging manifestations (Lempesis et al., 2024), confirmation requires PCR-based microbiological testing. Consequently, current guidelines emphasize that imaging should serve as a tool for assessing disease severity and monitoring treatment response rather than as a standalone diagnostic method for etiology identification (Garin et al., 2022).

### 5.2 C. psittaci

#### 5.2.1 Serological diagnosis

Serological tests for *C. psittaci* infection include complement fixation tests (CFTs) (Sheng et al., 2025), enzyme-linked immunosorbent assays (ELISAs/EIAs) (Heijne et al., 2021), MIF and immunoblotting (IPA) (Chen et al., 2015; Ji and Xiao, 2025). These are widely used during outbreaks but can cross-react with other *Chlamydia* species. Single serum tests are insufficient for confirmation; acute and convalescent samples are needed. However, sample collection is tough and time-consuming (Nieuwenhuizen et al., 2018). MIF antibody testing is the most sensitive and specific but is only for specialized labs (Wreghitt, 1993). Direct immunofluorescent staining of respiratory secretions is also used but needs validation for sensitivity and specificity (Yang F. et al., 2022).

#### 5.2.2 Molecular biological detection

For *C. psittaci* pneumonia, PCR-based molecular diagnosis is a research hotspot due to its high sensitivity and specificity. Nucleic acid amplification tests have significant advantages over traditional serological antibody tests. However, their clinical use is still limited to suspected outbreak scenarios, and they have not yet been widely adopted on a large scale (Rane et al., 2018). Although PCR detection technologies for *C. psittaci* have been developed, their commercialization has lagged behind (Rane et al., 2018). A study based on multiplex PCR analysis revealed that the actual prevalence of this pathogen in pneumonia patients is higher than previously estimated epidemiologically, highlighting the potential of molecular diagnostics in disease surveillance (Rane et al., 2018). The introduction of real-time PCR technology has further enhanced diagnostic performance. For example, a real-time PCR method based on TaqMan probes can simultaneously detect *C. pneumoniae*, *C. psittaci*. It is suitable for various clinical sample types and can be adapted to open molecular diagnostic platforms, providing a flexible and standardized testing solution for clinical use (Opota et al., 2017).

To optimize diagnostic specificity, a species-specific real-time PCR technique for *C. psittaci* achieves 100% sensitivity and specificity, yielding a positive predictive value of 100% and a negative predictive value of 88% (Opota et al., 2015). This cost-effective, high-throughput method is particularly suitable for large-scale screening and rapid diagnosis during outbreaks (Opota et al., 2015). Additionally, multiplex TaqMan real-time PCR detection kits further integrate the diagnostic workflow, enabling simultaneous detection of *C. psittaci*, *C. pneumoniae*, and an internal control (Wolff et al., 2018). The consistency of these kits with previous clinical specimen test results reaches 100%, validating their reliability and reproducibility in complex samples (Wolff et al., 2018).

#### 5.2.3 Imaging examinations

The imaging features of *C. psittaci* pneumonia are characterized by multimodal pulmonary lesions, and its chest X-ray and CT manifestations have specific diagnostic value (Chu et al., 2020). Lesions are often distributed in secondary pulmonary lobules, peribronchovascular bundles, or subpleural areas, with diffuse involvement of the pulmonary parenchyma and interstitium. Typical manifestations include extensive consolidation and ground-glass opacities (Chu et al., 2020). Notably, necrosis, cavity formation, and tree-in-bud signs are rare in the imaging of this disease, which helps differentiate it from other types of pneumonia (Wu et al., 2023). In severe cases, 86.9% of patients show involvement of multiple lung lobes (≥2 lobes), and multilobar lesions are significantly correlated with disease severity, suggesting that they can serve as an indicator of severe disease. After timely treatment, most imaging abnormalities can resolve within 2 weeks (Deng et al., 2024). Chest X-ray abnormalities are as high as 90%, mainly presenting as patchy shadows, reticular infiltrates, and varying degrees of consolidation, often accompanied by air bronchograms, with pleural effusion visible in some cases (Shi et al., 2021). CT imaging further reveals characteristic changes in severe cases, such as extensive bilateral pneumonia with pleural effusion or large areas of shadowing. Such lesions can gradually resolve within 2–4 weeks after drug treatment (Rybarczyk et al., 2020). In the early stages of the disease, it is often limited to a single lobe of the right lung, presenting as local inflammatory exudation and consolidation. As the disease progresses, it can spread to both lungs and develop pleural effusion. Only a few patients are left with pulmonary interstitial changes (Yang F. et al., 2022). Quantitative CT analysis shows that 94.3% of patients have bilateral or multiple patchy shadows, 68.6% have pleural effusion (54.3% bilateral), 51.4% have pleural thickening, 31.4% have pericardial effusion, and 40.0% have mediastinal lymphadenopathy. Among them, 51.4% of cases meet the criteria for severe pneumonia, and the disease progresses rapidly (Chen J. et al., 2024). Although these imaging features overlap with atypical pneumonia, their combined patterns and dynamic evolution still provide important evidence for clinical diagnosis.

#### 5.2.4 Metagenomic next-generation sequencing

The diagnosis of *C. psittaci* pneumonia is limited by the lack of rapid and accurate methods, resulting in fewer clinical reports, particularly of primary cases (He et al., 2023). In recent years, metagenomic next-generation sequencing (mNGS), through sequencing technology and data analysis, has enabled rapid identification of a wide range of pathogens, including bacteria, fungi, and viruses, significantly improving the detection rate of *C. psittaci*. A retrospective study of 27 severe cases, all diagnosed by BALF-based mNGS, demonstrated its clinical utility (Yang F. et al., 2022). This technology can cover rare pathogens without the need for specific amplification. By comparing clinical specimens with databases, mNGS has become a core method for *C. psittaci* detection (Deng et al., 2024). Clinical practice has shown that mNGS can provide etiological evidence within 24–48 hours, guiding timely adjustments to tetracycline-based targeted therapy, shortening ICU stay, then improving body temperature and oxygenation within 3–5 days, reducing the inappropriate use of broad-spectrum antibiotics and antifungal drugs, and thereby lowering the risk of resistance and hospitalization costs (Yang L. et al., 2019; He et al., 2023).

Despite the broad-spectrum detection advantages of mNGS, its limitations include high costs, low detection rates for intracellular bacteria, and missed diagnoses of influenza viruses, as well as the lack of standardized guidelines for report interpretation (Qu et al., 2022; Schlaberg et al., 2017; Li et al., 2021). Currently, research on targeted next-generation sequencing (tNGS) for *C. psittaci* remains limited (Zhang et al., 2023). Therefore, it is recommended that for patients with SCAP, especially those unresponsive to empirical treatment or with a history of special exposure, early combined mNGS testing should be performed to improve diagnostic timeliness and prevent disease progression (He et al., 2023).

### 5.3 C. trachomatis

#### 5.3.1 Laboratory tests

In traditional diagnostic methods, cell culture remains the “gold standard” (with specificity close to 100%), but its sensitivity is low(less than 80% for genital tract samples, and even lower for nasopharyngeal samples), time-consuming (3–7 days), and dependent on specialized laboratories, which limits its clinical practicality (Chandran and Boykan, 2009). Additionally, clinical studies using binary regression analysis have found that congenital heart disease, mixed infections, white blood cell counts exceeding 15, 000 cells/dl, and elevated PaCO_2_ are independent predictive factors for severe *C. trachomatis* pneumonia in children, providing a basis for early identification of high-risk patients (Chen W. et al., 2024). In summary, laboratory diagnosis needs to combine traditional methods with new molecular technologies to balance accuracy, timeliness, and cost-effectiveness, thereby optimizing the diagnosis and treatment strategies for *C. trachomatis* pneumonia.

#### 5.3.2 Serological diagnosis

Serological testing for *C. trachomatis* pneumonia is clinically valuable but must consider technical performance and clinical context. A cross-sectional study using ELISA to detect IgM antibodies found a 5.4% prevalence of *C. trachomatis* infection in infants under six months with lower respiratory tract infections (LRTIs) and linked infection to prematurity and low birth weight (Souza et al., 2012). Comparing traditional serological methods like MIF and commercial ELISA, SeroCT for *C. trachomatis* IgG detection showed 100% specificity, outperforming SeroCP’s low specificity (38.5%) for *C. pneumoniae*. This suggests high-specificity ELISA is key to reducing cross-reactions (Frikha-Gargouri et al., 2008). While whole-organism antigen-based ELISA has low sensitivity (51.5%–64.8%) and cross-reactivity issues, novel peptide antigen ELISA, using 5–11 specific B-cell epitope peptides, boosted sensitivity to 86.5%–91.8% (98% specificity), greatly improving serological diagnosis (Rahman et al., 2018). Additionally, the dual recombinase-assisted amplification (RAA) assay, targeting specific gene sequences of *C. trachomatis* and Mycoplasma pneumoniae, enables high-sensitivity (detection limit of 10 copies/μL), high-specificity, and rapid testing (within 30 minutes), making it ideal for on-site screening in resource-limited areas (Nie et al., 2022). However, current serological tests have limitations. IgG or IgA antibodies can’t alone confirm active infection and require clinical correlation to rule out cross-reactivity (Frikha-Gargouri et al., 2008). Thus, serological diagnosis should be combined with molecular testing, and efforts should continue to develop high-specificity antigens to enhance test performance.

Additionally, antigen-based (AD) rapid on-site testing (POCT) is widely used in low- and middle-income countries due to its low cost. However, its sensitivity is low (e.g., the combined sensitivity for cervical, vaginal, and male urine samples is 53%, 37%, and 63%, respectively), and it is not recommended as a screening test (Kelly et al., 2017). Studies have shown that there is no significant difference in diagnostic accuracy between different sample types (e.g., cervical and vaginal swabs) for AD-based POCT (Zhou Y. et al., 2021). However, first-void urine samples show higher sensitivity, suggesting that future improvements in sample collection devices may enhance test performance. New lateral flow immunoassay kits (such as the Qcare *Chlamydia* TRF kit) have shown good sensitivity and specificity, but further clinical validation is still needed (Zhou Y. et al., 2021). It is worth noting that the pooled sensitivity of antigen-based POCT in high-income countries is slightly higher than in low-income countries, although the difference is not statistically significant. This reflects the impact of healthcare service disparities on test performance and highlights the need for more real-world studies to assess its applicability (Zhou Y. et al., 2021).

#### 5.3.3 Molecular biological detection

In the nucleic acid amplification test (NAAT) detection of *C. trachomatis* pneumonia, PCR-based methods are considered the gold standard for clinical diagnosis due to their high specificity and sensitivity (Eboigbodin, 2019a). However, PCR-based methods’ reliance on complex equipment, time-consuming procedures, and the need for prior genetic material extraction limit their application in resource-limited settings (Eboigbodin, 2019a). To address this bottleneck, emerging isothermal amplification technologies such as loop-mediated isothermal amplification (LAMP) optimize sample processing steps by combining antimicrobial peptide lysates, enabling direct detection of *C. trachomatis* in urine samples (Eboigbodin, 2019a). This method offers advantages such as rapidity (completed within 1 hour) and low equipment requirements, making it suitable for point-of-care or primary care environments (Eboigbodin, 2019a). Worth mentioning is that while combination testing strategies have been proposed to improve diagnostic accuracy in resource-limited settings, recent evidence from 2025 indicates that combining DIF, rapid tests, and serology does not surpass the diagnostic performance of NAAT-based methods (Tošić-Pajić et al., 2025). The extended Youden index for optimal test combinations (RT/IgA: 94.6%) remains inferior to NAAT-POCT performance (sensitivity 95%, specificity 100%). Therefore, efforts should prioritize expanding NAAT accessibility rather than optimizing suboptimal test combinations (Tošić-Pajić et al., 2025). Similarly, strand invasion-based amplification (SIBA) technology achieves high sensitivity (detection limit of 10 copies/reaction) and specificity under isothermal conditions through multiplex detection design (mSIBA), and can be operated on portable instruments, providing a new strategy for rapid screening (Eboigbodin and Hoser, 2019). Real-time multiplex qPCR technology enhances detection efficiency and reduces cross-contamination risks by simultaneously amplifying *C. trachomatis* targets in a single reaction tube (Eboigbodin, 2019b). Clinical cases have shown that *C. trachomatis* pneumonia in a 9-week-old infant can be diagnosed through positive urine NAAT results combined with clinical manifestations (Robinson et al., 2014). Although a positive result cannot directly confirm lung infection, it provides new insights for simplifying sample collection (avoiding the need for nasopharyngeal swabs) and epidemiological research (Robinson et al., 2014). Currently, further validation of the sensitivity and specificity of urine NAATs and optimization of standardized protocols for isothermal amplification technologies are needed to promote the precision and accessibility of *C. trachomatis* pneumonia diagnosis.

Moreover, NAAT-based POCT has shown excellent performance in terms of sensitivity and specificity, with a combined sensitivity and specificity of 95% and 100% in urine samples, and 93% and 99% in rectal swabs, significantly outperforming traditional antigen detection (Kelly et al., 2017; Zhou Y. et al., 2021). Commercially available NAAT-POCT (such as the Cepheid GeneXpert CT/NG test) meets the minimum requirements of the WHO Target Product Profile (TPP), but its operation time still needs optimization (Zhou Y. et al., 2021). Some new molecular POC diagnostic technology (such as the io CT/NG Assay and Visby Medical Sexual Health Test) have made progress in speed and accuracy, potentially revolutionizing the rapid diagnosis of sexually transmitted infections (Zhou Y. et al., 2021). Studies have pointed out that defining the “gold standard” for infection through a combination of multiple NAATs (such as at least three positive results out of three NAATs or positive results from two samples) can significantly improve the reliability of sensitivity and specificity estimates (Martin et al., 2004). Although rapid NAAT can be used as a standalone test and shorten treatment time (e.g., the GeneXpert test has a sensitivity of 97.5%–98.7% and specificity of 99.4%–99.9%), its cost and equipment requirements limit its widespread use in resource-poor areas (Brook, 2015).

Finally, the most significant breakthrough in recent years has emerged in the realm of at-home self-testing. The FDA has granted marketing authorization enabling consumers to purchase over-the-counter test kits for sexually transmitted infections (including *C. trachomatis*) that can be self-sampled and self-administered in the home setting (Morris et al., 2021).

#### 5.3.4 Imaging examinations

The imaging features of *C. trachomatis* pneumonia have important diagnostic implications in infants and young children. Typical manifestations include a diffuse miliary reticulonodular pattern on chest X-ray or chest computed tomography (CT), which should be included in the differential diagnosis for infants hospitalized with respiratory symptoms (Olatunji et al., 2024). Chest X-ray, as a common screening tool, has a sensitivity of 100%, specificity of 73%, negative predictive value of 100%, and positive predictive value of 80%. It is highly reliable, especially in ruling out non-infectious causes (Olatunji et al., 2024). Studies show that 50% (15 cases) of 30 confirmed patients have hyperinflated lungs on chest X-ray, suggesting possible bronchiolitis or airway hyperresponsiveness (Camlar et al., 2009). Although imaging manifestations (such as miliary lesions) are not directly related to *C. trachomatis* colonization or infection, perinatal factors such as delivery mode and birth weight, their negative predictive value can effectively assist in ruling out the diagnosis (Olatunji et al., 2024). In clinical practice, combining serological or nucleic acid amplification tests with radiological features can improve the accuracy of identifying atypical pneumonia, especially when rapid microbiological evidence is unavailable (Chiang et al., 2005) (**Table 2**).

**Table 2:** Diagnostic methods of chlamydial pneumonia.

Diagnostic method	*C. pneumoniae* pneumonia	*C. psittaci* pneumonia	*C. trachomatis* pneumonia
Laboratory Tests/Culture	Cell culture (HEp-2/HL cell lines)	Cell Culture	Cell Culture
Serological Diagnosis	Microimmunofluorescence (MIF)	Microimmunofluorescence (MIF)	Peptide-based ELISA (High Specificity)
Molecular Diagnostics	PCR (Preferred Method)	Specific PCR (e.g., Real-time PCR)	Nucleic Acid Amplification Test (NAAT)
Imaging Studies	Chest X-ray/CT (Suggestive, non-specific)	Chest X-ray/CT (Characteristic findings)	Chest X-ray/CT (Significant in infants)
Other Key Technologies	Multiplex PCR Panels Metagenomic	Next-Generation Sequencing (mNGS)	Loop-mediated isothermal amplification (LAMP)

## 6 Treatment

### 6.1 Conventional antibiotic therapy

*C. pneumoniae* has unique biological features. Its cell wall includes an inner and outer membrane but has little peptidoglycan, which gives it natural resistance to β-lactam antibiotics. Outside a host, it exists as small, dense elementary bodies (EBs) (Garin et al., 2022). When entering respiratory mucosal epithelial cells, it transforms into metabolically active reticulate bodies (RBs) that multiply and release new EBs to infect nearby cells. *In-vitro* data indicate that *Chlamydia* can establish persistent infection and may contribute to chronic disease (Garin et al., 2022). In cell-culture assays, macrolides, tetracyclines and fluoroquinolones all exhibit activity. Clarithromycin achieves a minimum bactericidal concentration required to kill 90% of organisms (MBC_90_) against 49 strains that is significantly lower than that of erythromycin, whereas the fluoroquinolone sparfloxacin displays activity comparable to clarithromycin. Despite high *in-vitro* susceptibility, persistent pathogen carriage can still be observed in clinical throat-swab specimens (Garin et al., 2022). Although macrolides, cell-cycle inhibitors and fluoroquinolones demonstrate favorable *in-vitro* activity, bacteriological treatment failures continue to be reported, and unresolved issues in clinical, epidemiological and therapeutic domains demand further investigation (Gaillat, 1996). Meanwhile, evidence indicates that tetracyclines, erythromycin and earlier quinolones exert limited efficacy against *C. pneumoniae*, whereas the azalide azithromycin and the macrolide clarithromycin display superior *in-vitro* activity and are considered first-line options, yet microbiological relapse can still occur after prolonged courses (Cook and Honeybourne, 1995; Zele-Starcević et al., 2004). In addition, newer fluoroquinolones, tetracyclines and macrolides show *in-vivo* efficacy. Pediatric regimens comprise erythromycin 50 mg/kg/day for 10–14 days, clarithromycin 15 mg/kg/day for 10 days, or azithromycin 10 mg/kg on day 1 followed by 4 mg/kg daily for 4 days, with extension occasionally required to achieve pathogen eradication (Wise et al., 1992; Kauppinen and Saikku, 1995; Hammerschlag, 2003).

Management of *C. psittaci* pneumonia is stratified by disease severity. In non-severe patients, tetracyclines (such as doxycycline or minocycline) and fluoroquinolones (including moxifloxacin) all exert rapid therapeutic effects. Following appropriate treatment, fever declines within 24 h and is controlled within 48 h, and pulmonary lesions resolve within approximately 2 weeks. Doxycycline remains the preferred agent, while tetracyclines, fluoroquinolones, and macrolides are all acceptable therapeutic options (Zhang A. et al., 2022; He et al., 2023; Deng et al., 2024). Mild-to-moderate disease is typically treated with oral doxycycline or minocycline. For patients with tetracycline contraindications, macrolides such as azithromycin are regarded as the optimal substitute (Katsura et al., 2020), and fluoroquinolones, such as moxifloxacin and levofloxacin, have also demonstrated favorable efficacy. For critically ill patients, early screening and prompt, targeted therapy is essential, as these patients frequently require intensive care unit (ICU) admission, mechanical ventilation, and prolonged hospitalization (Deng et al., 2024). When such patients present with high fever, cough, scant sputum, no substantial increase in leukocyte or neutrophil counts, and concomitant elevations in serum creatinine and aminotransferases, and when conventional β-lactam antibiotics remain ineffective within 24–48 h*, C. psittaci* infection should be strongly suspected (Deng et al., 2024). Metagenomic next-generation sequencing (mNGS) of bronchoalveolar lavage fluid can then offer critical diagnostic evidence (Deng et al., 2024). For critically ill patients, intravenous doxycycline is recommended for a minimum of 14 days and preferably extended to 21 days to prevent relapse (Balsamo et al., 2017). In some cases, combination therapy with tetracyclines, macrolides and fluoroquinolones is required to manage secondary bacterial infections and other complications. At the same time, attention should be paid to the issue of tetracycline resistance, which may originate from the environment and is now widely distributed in both commensal bacteria and pathogens. Clinically, dehydrotetracycline or similar agents can be considered to inhibit Tet enzyme activity and thus maintain drug efficacy (Thaker et al., 2010; Markley et al., 2019; Gasparrini et al., 2020). If the patient’s condition deteriorates, the regimen should be re-evaluated. Adding tigecycline may improve respiratory failure and overall prognosis (Kong et al., 2021; Shi et al., 2021).

Treatment protocols for *C. trachomatis* pneumonia are primarily based on rational antibiotic selection and clinical guideline recommendations. The Centers for Disease Control and Prevention (CDC) lists azithromycin and doxycycline as first-line agents (Zele-Starcević et al., 2004). Erythromycin, ofloxacin and levofloxacin serve as alternatives. Although *in-vitro* studies have documented rising antibiotic resistance among *C. trachomatis* isolates, the clinical significance of these findings remains unclear (Zele-Starcević et al., 2004). In infants, erythromycin has demonstrated clear efficacy. A study of thirty pediatric cases of *C. trachomatis* pneumonia reported that all patients responded well to erythromycin, with clinical improvement achieved after a mean of 3.53 days and no deaths recorded (Chiang et al., 2005). In preterm infants, prophylactic macrolide therapy (e.g., erythromycin) targeting nasopharyngeal *C. trachomatis* may reduce the risk of *Chlamydia*-associated pneumonia but does not prevent pneumonia caused by other pathogens (Bekler et al., 2012). For special populations such as neonates with renal impairment or congenital heart disease, intravenous azithromycin is particularly recommended. Its use is guided by specific protocols that outline indications (e.g., *C. trachomatis* pneumonia), provide age-stratified dosing, and include strategies for managing adverse events (Zhou P. et al., 2021). Systemic erythromycin is widely recommended for *infantile C. pneumonia*, underscoring the importance of early intervention to lower the risk of chronic lung disease (Farzam et al., 2025). Antimicrobial resistance must be continuously monitored. Current regimens remain centered on high clinical efficacy and are optimized through individualized prescribing (**Table 3**).

**Table 3:** Conventional antibiotic therapy for pneumonia caused by *Chlamydia* species.

Pathogen	First-line agents	Alternative agents	Key considerations
*C. pneumoniae*	Azithromycin, Clarithromycin	Doxycycline, Fluoroquinolones (e.g., Levofloxacin)	Macrolides are preferred in children. Microbiological relapse can occur.
*C. psittaci*	Doxycycline	Azithromycin, Moxifloxacin	Tetracyclines are contraindicated in children < 8 years. Treat for 14–21 days (longer for severe cases).
*C. trachomatis*	Azithromycin, Doxycycline	Levofloxacin, Erythromycin	Erythromycin is the primary choice for infants and neonates.

### 6.2 Emerging targeted and non-antibiotic therapies

Although conventional antibiotics remain the cornerstone for treating CAP caused by *C. pneumoniae*, research over the past five years has revealed complexities in their application and future alternative pathways. First-line recommended agents include doxycycline, azithromycin, and levofloxacin (Tagini et al., 2025). However, the notion that *C. pneumoniae* does not exhibit clinically relevant antibiotic resistance has been disproven. Recent studies have confirmed that resistance to fluoroquinolones is associated with specific mutations in the gyrA gene (Tong et al., 2025). Due to extreme difficulty in cultivation, routine susceptibility testing is rarely performed, potentially allowing resistant strains to spread undetected in populations; thus, “absence of resistance reports” cannot be simply equated with “absence of risk” (Sandoz and Rockey, 2010; Kohlhoff and Hammerschlag, 2015). Furthermore, the causes of treatment failure are more complex. In addition to resistance, antibiotic-induced entry into a metabolically quiescent “persistent state” represents a significant factor, during which bacteria become insensitive to antibiotics and require prolonged courses for eradication (Borel et al., 2016; Tagini et al., 2025). Given these challenges, along with the global resistance crisis driven by overuse of broad-spectrum antibiotics, novel therapeutic strategies targeting *Chlamydia* are gaining increasing attention. Distinct from these approaches, recent high-throughput screening has uncovered a promising new target: the FabH enzyme essential for chlamydial fatty acid biosynthesis (Ölander et al., 2025). Over 60 chemically diverse compounds have been identified that selectively inhibit *Chlamydia* growth without harming host cells. The most potent candidates covalently bind to the FabH active site, offering a novel mechanism for selective treatment that bypasses traditional resistance pathways (Ölander et al., 2025). One class comprises non-antibiotic compounds targeting the chlamydial developmental cycle itself, exemplified by JO146, which act by interfering with essential proteins such as the HtrA serine protease (Hou et al., 2022). Another class includes natural products, such as tea polyphenols and rutin and other polyphenolic compounds, which demonstrate *in vitro* activity but often require high concentrations, limiting their clinical application (Hou et al., 2022). The most promising direction is host-directed therapy (HDT), which combats infection by enhancing host defense capabilities rather than direct bactericidal action, thereby offering potential to fundamentally address resistance issues (Liu et al., 2024; Shapira et al., 2024). Specific strategies include: inducing autophagy to eliminate intracellular bacteria (Lorente-Torres et al., 2024; Yang S. et al., 2024); modulating inflammatory responses, such as combined use of NLRP3 inflammasome inhibitors or IL-1β antagonists, which can control infection while attenuating tissue damage (Tumurkhuu et al., 2018); and inhibiting bacterial growth by inducing cytokines such as IFN-γ to deplete essential nutrient tryptophan within host cells (Hou et al., 2022). These non-antibiotic therapies represent a fundamentally different therapeutic philosophy, offering diverse and innovative solutions for future management of chlamydial infections (**Supplementary Figure 1**).

## 7 Prevention

However, in the past five years, significant progress has been made in candidate antigens and vaccine platforms, bringing new hope for future vaccine development (Huang et al., 2025b). MOMP has been the most widely studied candidate antigen because it can trigger neutralizing antibodies and protective CD4^+^ T cell responses (Hickey et al., 2009; McEntee et al., 2020; Collar et al., 2025). In addition, several other proteins have also been confirmed to have immunogenicity and become new candidate targets, including outer membrane protein 2 (Omp2), heat shock protein 60 (Hsp60), chlamydial outer membrane protein N (CopN), and polymorphic membrane protein D (PmpD), etc (Penttilä et al., 2004; Tammiruusu et al., 2007; Saleki et al., 2022; Collar et al., 2025). In terms of vaccine technology platforms, the research trend is shifting from traditional inactivated or attenuated vaccines to more advanced strategies, such as using bacterial outer membrane vesicles (OMVs) as delivery platforms or applying DNA or mRNA technologies to vaccine development (Penttilä et al., 2000; Gerritzen et al., 2017; De Meyst et al., 2025). These new technologies are expected to induce stronger and more durable immune responses. Although the challenges remain severe, the research pipeline is no longer empty. A candidate vaccine CTH522 for *C. trachomatis* has entered phase I clinical trials (NCT03562225), evaluating its safety and immunogenicity in humans (Hou et al., 2022). Studies in animal models have also provided valuable data, such as nasal or percutaneous immunization showing good protective effects in mouse models (Skelding et al., 2006). In summary, although it will take some time for an ideal *chlamydia* vaccine to be developed, this field has shifted from past stagnation to a promising new stage, with new scientific discoveries and technological advancements continuously driving vaccine development forward.

## 8 Conclusions and perspectives

We comprehensively explore the roles of three *Chlamydia* species, *C. pneumoniae*, *C. psittaci*, and *C. trachomatis*, in community-acquired pneumonia (CAP). These pathogens collectively impose a significant public health burden due to their diverse transmission routes and nonspecific clinical presentations, which often result in delayed diagnosis and increased risks of chronic complications. While advances in diagnostic technologies have improved our understanding of these infections, limitations such as the low sensitivity of traditional serological assays for chronic infections suggest that the true incidence of these pathogens is likely underestimated, posing challenges for healthcare resource allocation and prevention strategies. To address these gaps, we systematically evaluate the epidemiological characteristics, clinical manifestations, pathogenic mechanisms, diagnostic approaches, and treatment strategies for chlamydial pneumonia.

Moving beyond established diagnostic and treatment paradigms, the research then critically examines emerging challenges in antibiotic therapy, including fluoroquinolone resistance associated with gyrA mutations and the persistent state phenomenon, alongside innovative non-antibiotic therapeutic strategies targeting the chlamydial developmental cycle such as JO146, host-directed therapies including autophagy induction and NLRP3 inflammasome modulation, as well as natural products (Hou et al., 2022; Liu et al., 2024; Tagini et al., 2025; Tong et al., 2025). Furthermore, the survey highlights transformative progress in vaccine development, covering candidate antigens from MOMP to novel targets such as PmpD, technological evolution to advanced platforms including OMV-based and mRNA vaccines, and the transition to first-in-human clinical trials exemplified by CTH522, underscoring the field’s shift from historical stagnation to a promising new era (Gerritzen et al., 2017; Hou et al., 2022; Collar et al., 2025).

Despite these comprehensive insights, several broader challenges remain for the field at large. The clinical translational gap between promising preclinical non-antibiotic therapies and their practical application requires urgent attention, as does the integration of novel therapeutic strategies with existing antibiotic regimens to optimize patient outcomes. While rapid molecular diagnostics have advanced, their global accessibility and cost-effectiveness need improvement. The long-term consequences of chronic *C. pneumoniae* infection, including potential associations with chronic diseases, warrant deeper investigation. Addressing these overarching issues will require continued interdisciplinary collaboration to translate scientific advances into tangible clinical benefits for patients worldwide.
