# Supplementary figures and images for: Chlamydia beyond the genital tract: a major contributor to community-acquired pneumonia
Source: Front Cell Infect Microbiol. 2026 May 8;16:1787885. doi: 10.3389/fcimb.2026.1787885 (PMC13195017; doi:10.3389/fcimb.2026.1787885)

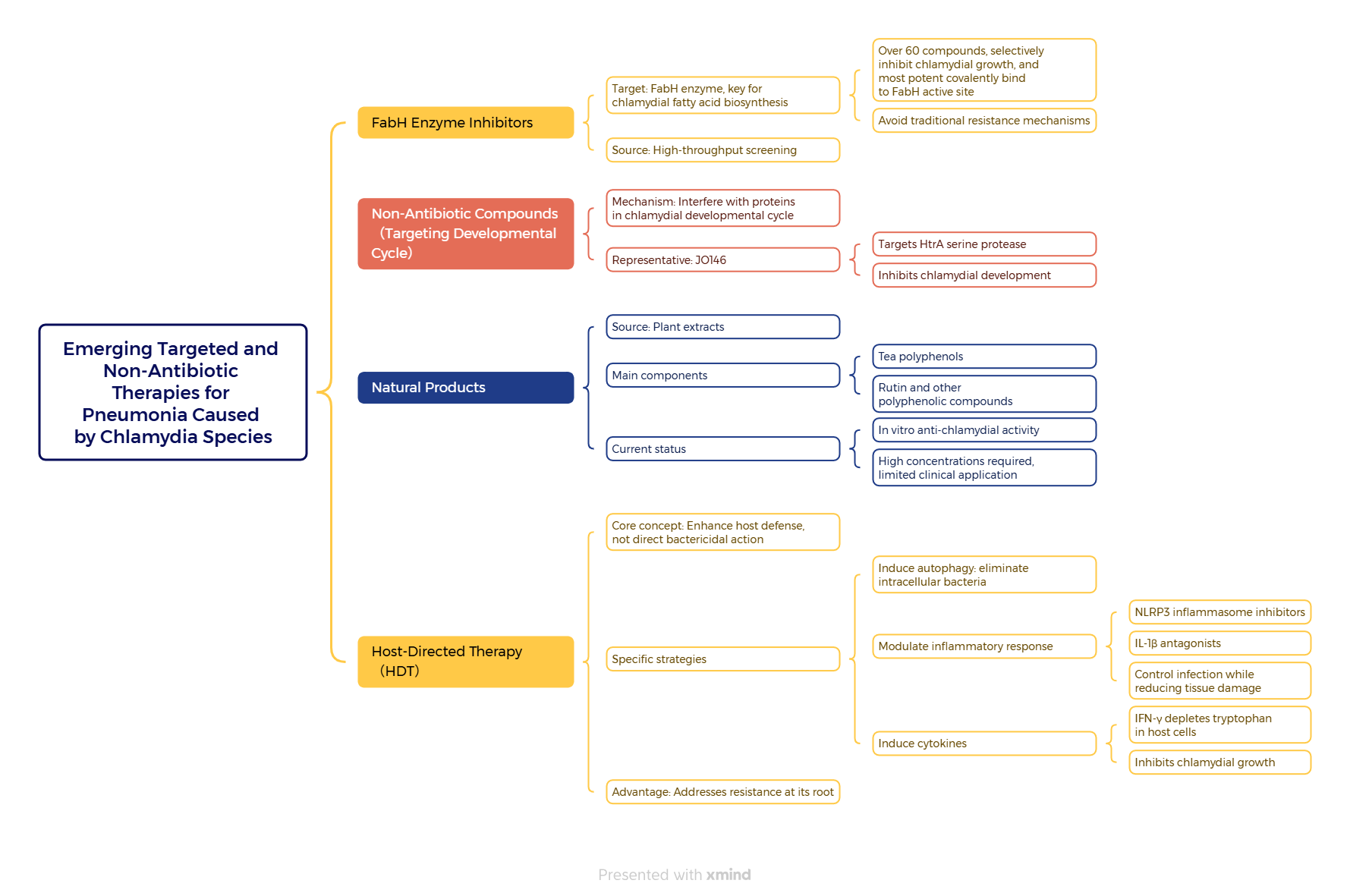

Supplement: Supplementary file 1 [file Image1.tif]
